# Perspectives on Aging Well Among Older Black Women in the US

**DOI:** 10.1093/geroni/igab046.3161

**Published:** 2021-12-17

**Authors:** Jacquelyn Coats

**Affiliations:** Brown School, Washington University in St. Louis, Saint Louis, Missouri, United States

## Abstract

Despite changes in the demography of older Black women who are living longer, there is limited research on how older Black women conceptualize and understand successful and healthy aging. The objective of this presentation is to interrogate the meaning and cultural aspects of aging among older Black women, gaining insight into how gender and race operate and intersect to shape experiences and perceptions of aging. Using an intersectionality framework, this qualitative study was conducted with three older Black women. The women ranged in age from 58-65 years old, each residing in an US urban city (Detroit, St. Louis, Atlanta). Data were collected between October and November 2020, using a semi-structured, open-ended interview protocol to encourage participants to provide in-depth descriptions of how they conceptualized aging. Interviews were between 2 – 2.5 hours, conducted via videoconferencing, and audio-recorded for transcription. Participants discussed: the life experiences that have shaped their ability to age well; what it means to age well, and factors that might hinder someone from aging well. Transcripts were coded using a constructivist grounded theory approach. Results revealed six themes Black women in later life identify: aging well, aging as a mindset, independence and freedom, authenticity, personal control and preparation, and aging role models. This extends the knowledge base on how older Black women view aging and factors that enhance or diminish their ability to age well. Results from this study can be used to enhance the development of public health and social work interventions with older Black women.

